# Integrating preoperative imaging and clinical factors: a nomogram for predicting functional outcomes after rotator cuff repair

**DOI:** 10.3389/fsurg.2026.1779971

**Published:** 2026-04-13

**Authors:** Zhihao Liao, Sheng Chen, Yuke Song, Weipeng Zheng, Zhijun Liu, Suming Zheng, Hewei Wei

**Affiliations:** Department of Sports Medicine, The Third Affiliated Hospital of Guangzhou University of Traditional Chinese Medicine, Guangzhou, Guangdong, China

**Keywords:** functional outcome, nomogram, prognostic factors, radiomics, rotator cuff repair

## Abstract

**Objective:**

The predictive value of a nomogram model constructed by integrating radiomics features and clinical risk factors for the functional outcomes of patients after rotator cuff repair was evaluated.

**Methods:**

A total of 367 patients who underwent rotator cuff repair from January 2021 to December 2023 were selected. Pre - operative shoulder MRI images were collected and radiomics features were extracted, and clinical baseline data were also collected. The patients were randomly divided into a training set (*n* = 257) and a validation set (*n* = 110) at a ratio of 7:3. In the training set, univariate analysis was used to identify factors associated with postoperative functional outcomes, which were evaluated by the Constant-Murley score at 12 months after surgery and classified into good or poor categories. Least absolute shrinkage and selection operator (LASSO) regression was used for radiomics feature dimensionality reduction and variable screening, and then independent predictive factors were identified by multivariate Logistic regression. A nomogram model was established accordingly. The area under the receiver operating characteristic curve (AUC), calibration curve, and decision curve analysis (DCA) were used to evaluate the discrimination, calibration, and clinical utility of the model, respectively.

**Results:**

Multivariate analysis showed that age, pre - operative visual analog scale score, tear area, tear maximum length, tendon retraction distance, standard deviation of gray - scale, and entropy of the gray - level co - occurrence matrix were independent predictive factors for poor postoperative functional outcomes in patients undergoing rotator cuff repair (*P* < 0.05). The AUCs of the nomogram model developed based on these factors were 0.817 (95% CI: 0.750–0.883) in the training set and 0.721 (95% CI: 0.600–0.843) in the validation set, respectively. The calibration curve showed good consistency between the predicted probability and the actual risk.

**Conclusion:**

The nomogram model integrating radiomics features and clinical factors has potential utility in predicting functional outcomes after rotator cuff repair, and may thus provide a valuable reference for clinical individualized treatment and prognosis assessment.

## 1 Introduction

Rotator cuff injury is a common shoulder disorder in orthopedics and sports medicine, and its prevalence is rising with population aging and an increase in sports-related injuries. It severely impairs shoulder joint mobility and patients’ quality of life (1, 2). Rotator cuff repair is the core surgical intervention for moderate-to-severe rotator cuff injuries, which effectively reconstructs the anatomical structure and mechanical stability of the rotator cuff tendon. However, significant individual differences in postoperative functional recovery are observed in clinical practice even among patients receiving standardized surgical treatment (3, 4). Some patients suffer from residual shoulder pain and limited mobility postoperatively, while others achieve favorable joint function recovery. This variability is associated not only with surgical techniques but also with patient baseline status and injury characteristics. Thus, accurate prediction of postoperative functional outcomes has become a key clinical challenge.

Currently, clinical prognosis assessment after rotator cuff repair mainly relies on traditional clinical indicators, which have limited predictive value. Traditional imaging assessments are also limited to qualitative descriptions, only identifying the tear location and approximate range. These shortcomings hinder clinicians from conducting individualized and accurate prediction of postoperative functional outcomes, thereby affecting the optimization of treatment plans and rehabilitation programs (5, 6). In recent years, radiomics technology has been increasingly applied in orthopedics. Studies have confirmed that radiomics features derived from shoulder magnetic resonance imaging (MRI) can effectively assess the severity of rotator cuff tendon injury, fatty infiltration grade, and fibrosis level, providing a novel perspective for preoperative assessment and prognostic prediction (7, 8).

As a visual prediction tool, the nomogram can transform the results of a multi - factor regression model into an intuitive scoring system, enabling clinicians to rapidly assess the prognosis risk of individual patients. It is widely applied in the field of disease prognosis prediction (9–11). Against this background, the present study aims to overcome the limitations of traditional single - indicator prediction, integrate radiomics features of preoperative shoulder MRI and clinical risk factors, and develop a novel nomogram model for predicting the functional outcomes after rotator cuff repair.

## 2 Materials and methods

### 2.1 Sample size estimation

Referring to the empirical rule that the sample size in multi - factor regression analysis should be 10 - 20 times the number of variables included in the model, this study plans to include about 10–12 potential predictive variables, including radiomics features and clinical risk factors. Initially, it is estimated that at least 100–240 samples are needed. Secondly, considering the influence of the number of outcome events on the model stability, the incidence of poor functional outcomes after rotator cuff repair in clinical practice is about 25%—30% (12–14). According to the requirement that the number of outcome events should be at least 5–10 times the number of variables, the number of poor outcome events should reach 50–120, corresponding to a total sample size of 200–320. Meanwhile, since the study needs to divide the samples into a training set and a validation set at a ratio of 7:3 and reserve a 10% sample dropout rate to account for follow - up losses, the final total sample size required for this study is determined to be 320. In fact, 367 patients who met the inclusion and exclusion criteria from January 2021 to December 2023 were included, with 257 cases in the training set and 110 cases in the validation set. There were 77 cases of poor functional outcome events in the training set and 31 cases in the validation set. Both the sample size and the number of events meet the statistical requirements for model construction and validation, thus ensuring the reliability and robustness of the nomogram model.

### 2.2 Research subjects

Patients who were admitted to the Department of Orthopedics and Sports Medicine of our hospital and underwent rotator cuff repair from January 2021 to December 2023 were retrospectively enrolled. This study was approved by the ethics committee of The Third Affiliated Hospital of Guangzhou University of Traditional Chinese Medicine (Approval No. PJ-KY-20230321-002), and all patients or their legally authorized representatives signed written informed consent forms in accordance with the Declaration of Helsinki. The diagnostic and severity grading criteria for rotator cuff injury referred to the “Clinical Practice Guidelines for Rotator Cuff Injury” issued by the American Academy of Orthopaedic Surgeons (AAOS) (15).

#### 2.2.1 Inclusion criteria

(1) Age ≥18 years. (2) Diagnosed with rotator cuff injury through clinical symptoms, physical examination, and shoulder MRI, and meeting the surgical indications for rotator cuff repair. (3) Completing shoulder MRI examination within 1 week before surgery, with clear imaging data available for radiomics feature extraction. (4) Having complete clinical baseline data including age, gender, injured side, rotator cuff tear size, etc, and obtaining the Constant - Murley functional score after at least 12 months of postoperative follow - up.

#### 2.2.2 Exclusion criteria

(1) Poor quality of preoperative MRI images, missing key clinical data, or incomplete postoperative follow - up, which may affect variable extraction and outcome assessment. (2) Comorbid with other shoulder joint diseases including the acute phase of subacromial impingement syndrome, glenoid labral tear, purulent arthritis, etc. (3) Comorbid with severe insufficiency of important organs such as the heart, liver, and kidneys, malignant tumors, coagulation disorders, or mental diseases, and unable to cooperate with treatment and follow - up. (4) With a history of shoulder joint surgery or contraindications for rotator cuff repair.

### 2.3 Research indicators and data collection

Demographic, clinical, and surgery - related indicators of the research subjects were collected through the electronic medical record system and preoperative assessment questionnaires, including age, gender, body mass index (BMI), smoking history, comorbidities of type 2 diabetes, comorbidities of primary hypertension, and comorbid osteoporosis. The degree of shoulder pain was evaluated by the visual analogue scale (VAS) before surgery, and the shoulder joint function status was evaluated by the Constant - Murley scoring scale. The type of rotator cuff tear (full - thickness/partial tear) and the surgical method (arthroscopic repair/open repair) were recorded during the operation. All clinical data were independently extracted and verified by two trained orthopedic surgeons, with a Kappa value of consistency >0.85.

### 2.4 Radiomics feature extraction pipeline

All radiomics features were extracted from the preoperative fat-suppressed T2-weighted imaging (FS-T2WI) sequences, which provided clear contrast between the injured tendon and surrounding tissues to ensure accurate feature calculation. Before feature extraction, the original MRI images were preprocessed with standardized steps strictly following the Image Biomarker Standardization Initiative (IBSI) preprocessing criteria: (1) voxel resampling was performed with linear interpolation to a uniform isotropic spatial resolution of 1 × 1 × 1 mm, which eliminated the influence of different scanner parameters and acquisition protocols on feature values; (2) min-max intensity normalization was applied to scale all gray values to a fixed range of 0–255 [calculated as: normalized value = (original value - minimum gray value)/(maximum gray value - minimum gray value) × 255], which standardized the gray value distribution across all images; (3) discretization of the normalized gray values was conducted using a fixed bin width of 25 (the bin width was determined based on the IBSI recommended range for musculoskeletal MRI texture analysis) to reduce the impact of random image noise on texture feature calculation.

Radiomics features were extracted using the pyradiomics package (version 3.0.1) in Python 3.9.13 (Anaconda 2022.10), and the feature extraction process was configured with the default IBSI-compliant parameters of the pyradiomics package. This package is fully compliant with the Image Biomarker Standardization Initiative (IBSI) guidelines, including strict adherence to IBSI specifications for feature naming, calculation algorithms, and preprocessing workflows, which ensures the standardization and reproducibility of feature calculation. A total of 128 radiomics features were extracted, including first-order statistics (e.g., mean gray value, standard deviation of gray values), second-order texture features (e.g., contrast and entropy based on the gray-level co-occurrence matrix, GLCM), and higher-order features.

All ROI delineation and radiomics feature extraction were performed by two attending radiologists with 5 years of experience in musculoskeletal imaging, who were completely blinded to the clinical outcomes (postoperative functional recovery status) of the patients to avoid subjective bias. The ROI delineation was conducted volumetrically on the FS-T2WI sequences: the radiologists manually outlined the 3D boundary of the injured tendon layer by layer, covering the entire injured area rather than a single representative slice. When discrepancies in ROI delineation or feature extraction results occurred between the two radiologists, a third senior associate chief radiologist with 10 years of experience was invited for arbitration, and the final results were determined based on the arbitration consensus. The intraclass correlation coefficient (ICC) was calculated to assess the inter-observer reliability, with ICC values >0.90 for all ROI measurements and >0.85 for all radiomics features, indicating good inter-observer consistency.

### 2.5 Outcome definition

The outcome event of this study was the shoulder joint functional recovery of patients 12 months after rotator cuff repair. The internationally recognized Constant - Murley shoulder joint function scoring scale was used for functional outcome assessment. This scale consists of four core dimensions: pain (15 points), daily activities (20 points), shoulder joint range of motion (40 points), and muscle strength (25 points), with a total score of 100 points. A higher score indicates better recovery of shoulder joint function. According to clinical practice and relevant research standards, the outcome event was specifically defined as two categories. Good functional recovery was defined as a Constant - Murley score ≥ 80 points, in which patients had no obvious shoulder pain and could normally meet their daily life and sports needs (16). Poor functional recovery was defined as a Constant - Murley score <80 points, where patients had persistent shoulder pain, limited mobility, or their daily life and sports functions were significantly affected. The confirmation of the outcome event was completed through on - site assessment during the 12 - month postoperative outpatient follow - up. Two orthopedic surgeons who did not participate in the surgery independently scored the patients. When the scoring results were inconsistent, a consensus was reached through joint review of imaging data and physical examination to ensure the objectivity and consistency of the outcome definition.

### 2.6 Statistical methods

In this study, SPSS 26.0 and R 4.5.1 software were used for data statistical analysis, and GraphPad Prism 9.0 was used for chart drawing. The test level *α* = 0.05, and a two - sided test with *P* < 0.05 was considered statistically significant. For measurement data, a normality test was first performed. If the data conformed to a normal distribution, they were expressed as X ± SD, and the t - test was used for comparison between groups. If the data did not conform to a normal distribution, they were expressed as the median (interquartile range), and the Mann - Whitney U test was used for comparison between groups. Count data were expressed as the number of cases (percentage), and the *χ*^2^ test was used for comparison between groups. Before grouping, the balance of the baseline clinical characteristics of the two groups was verified through the above - mentioned tests.

In the training set, the shoulder joint functional outcome 12 months after surgery was used as the dependent variable, and clinical risk factors were used as independent variables for univariate analysis to screen potential predictive indicators with *P* < 0.05. Notably, no missing data were found in all clinical and imaging indicators of the included patients after strict data collection and verification, so no missing data imputation or elimination was required. Least absolute shrinkage and selection operator (LASSO) regression analysis was performed using the glmnet package in R language for binary classification to conduct variable compression and redundancy elimination. The 5-fold cross-validation was used to traverse the regularization parameter lambda, and the optimal parameter was determined based on the lambda.min criterion, and the core predictive variables with non-zero regression coefficients were retained and included in the multi-factor Logistic regression model. The step-wise backward method was used to screen independent predictive factors for the functional outcome after rotator cuff repair. Before constructing the regression model, the variance inflation factor (VIF) was used to detect the multicollinearity among independent variables, and the variables with VIF < 2 were considered to have no significant multicollinearity. The R language rms package was used to construct the nomogram model. GraphPad Prism 9.0 was used to draw the receiver operating characteristic (ROC) curve, and the area under the ROC curve (AUC) and the 95% confidence interval (95% CI) were calculated to evaluate the model's discrimination. A calibration curve was drawn, and the Hosmer-Lemeshow test was used to evaluate the consistency between the predicted probability and the actual outcome. Internal validation was performed through 1,000-times non-parametric Bootstrap sampling (sampling with replacement from the training set), and the over-fitting bias was corrected by recalculating the C-index of the model in each sampling dataset to verify the stability and reliability of the prediction model.

## 3 Results

### 3.1 Comparison of basic characteristics between the training set and the validation set

Among the 257 patients in the training set, 77 cases (29.96%) had poor functional outcomes after rotator cuff repair. Among the 110 patients in the validation set, 31 cases (28.18%) had poor outcomes. There were no statistically significant differences in the general data between the training set and the validation set (*P* > 0.05) (Table 1).

**Table 1:** Comparison of basic characteristics between the training set and the validation set.

Indicators		Training set (*n* = 257)	Validation set (*n* = 110)	*t/χ^2^*	*P*
Age (years)		53.36 ± 8.61	52.34 ± 7.54	1.082	0.280
Gender	Male	145 (56.42)	57 (51.82)	0.659	0.417
	Female	112 (43.58)	53 (48.18)		
BMI (kg/m^2^)		23.62 ± 2.18	23.15 ± 3.01	1.709	0.088
Smoking history	Yes	78 (30.35)	31 (28.18)	0.173	0.677
	No	179 (69.65)	79 (71.82)		
Comorbid diabetes	Yes	32 (12.45)	15 (13.64)	0.097	0.756
	No	225 (87.55)	95 (86.36)		
Combined hypertension	Yes	55 (21.40)	25 (22.73)	0.080	0.778
	No	202 (78.60)	85 (77.27)		
Comorbid osteoporosis	Yes	37 (14.40)	18 (16.36)	0.234	0.629
	No	220 (85.60)	92 (83.64)		
Rotator cuff tear type	Full - thickness	95 (36.96)	37 (33.64)	0.371	0.543
	Partial	162 (63.04)	73 (66.36)		
Surgical approach	Arthroscopic repair	210 (81.71)	88 (80.00)	0.148	0.701
	Open repair	47 (18.29)	22 (20.00)		
Preoperative VAS		3.26 ± 1.36	3.52 ± 1.64	1.431	0.154
Preoperative Constant - Murley score		64.34 ± 7.73	65.54 ± 6.87	1.290	0.198
Tear area (mm^2^)		129.01 ± 32.38	132.54 ± 35.64	0.946	0.345
Tear maximum length (mm)		22.84 ± 5.87	23.58 ± 6.24	1.074	0.284
Tendon retraction distance (mm)		8.66 ± 2.66	8.45 ± 2.75	0.613	0.540
Supraspinatus muscle volume (cm^3^)		18.14 ± 3.16	17.54 ± 2.51	1.911	0.057
Infraspinatus muscle volume (cm^3^)		20.24 ± 3.63	20.54 ± 4.21	0.671	0.503
Mean gray level		149.81 ± 12.57	151.25 ± 10.05	1.097	0.274
Gray - level standard deviation		25.89 ± 4.41	26.54 ± 5.08	1.278	0.202
GLCM contrast		35.61 ± 6.32	36.14 ± 7.15	0.758	0.449
GLCM entropy		2.20 ± 0.38	2.28 ± 0.57	1.402	0.163
Long - run high - gray - level		12.28 ± 2.41	12.42 ± 3.10	0.433	0.665
Short - run low - gray - level		18.56 ± 3.20	17.66 ± 4.21	1.844	0.067

BMI, body mass index; VAS, visual analogue scale; GLCM, Gray-Level Co-occurrence Matrix.

### 3.2 Univariate analysis of influencing factors for functional outcomes after rotator cuff repair

Univariate analysis showed that in the training set, there were statistically significant differences in age, preoperative VAS, tear area, tear maximum length, tendon retraction distance, standard deviation of gray - scale, Preoperative Constant - Murley score and GLCM entropy between patients in the poor - outcome group and those in the good - outcome group (*P* < 0.05) (Table 2).

**Table 2:** Univariate analysis of poor outcomes after rotator cuff repair.

Indicators		Poor-outcome group (*n* = 77)	Good-outcome group (*n* = 180)	*t/χ^2^*	*P*
Age (years)		55.71 ± 9.26	52.35 ± 8.12	2.914	0.004
Gender	Male	40 (51.95)	105 (58.33)	0.894	0.344
	Female	37 (48.05)	75 (41.67)		
BMI (kg/m^2^)		23.78 ± 2.34	23.56 ± 2.12	0.774	0.440
Smoking history	Yes	28 (36.36)	50 (27.78)	1.881	0.170
	No	49 (63.94)	130 (72.22)		
Comorbid diabetes	Yes	12 (12.58)	20 (11.11)	0.990	0.320
	No	65 (84.42)	160 (88.89)		
Combined hypertension	Yes	20 (25.97)	35 (19.44)	1.367	0.242
	No	57 (74.03)	145 (80.56)		
Comorbid osteoporosis	Yes	15 (19.48)	22 (12.22)	2.305	0.129
	No	62 (80.52)	158 (87.78)		
Rotator cuff tear type	Full - thickness	35 (45.45)	60 (33.33)	3.401	0.065
	Partial	42 (54.55)	120 (66.67)		
Surgical approach	Arthroscopic repair	60 (77.92)	150 (83.33)	1.057	0.304
	Open repair	17 (22.08)	30 (16.67)		
Preoperative VAS		3.59 ± 1.56	3.12 ± 1.25	2.383	0.019
Preoperative Constant - Murley score		62.15 ± 8.21	65.28 ± 7.34	3.020	0.003
Tear area (mm^2^)		137.52 ± 35.68	125.36 ± 30.25	2.792	0.006
Tear maximum length (mm)		24.48 ± 6.75	22.15 ± 5.32	2.694	0.008
Tendon retraction distance (mm)		9.59 ± 3.42	8.25 ± 2.15	3.177	0.002
Supraspinatus muscle volume (cm^3^)		17.89 ± 3.25	18.25 ± 3.12	0.835	0.404
Infraspinatus muscle volume (cm^3^)		19.98 ± 3.78	20.36 ± 3.56	0.769	0.443
Mean gray level		148.78 ± 13.12	150.25 ± 12.34	0.852	0.395
Gray - level standard deviation		27.12 ± 4.56	25.36 ± 4.25	2.959	0.003
GLCM contrast		36.48 ± 6.75	35.25 ± 6.12	1.432	0.153
GLCM entropy		2.32 ± 0.45	2.15 ± 0.32	2.972	0.004
Long - run high - gray - level		12.59 ± 2.56	12.15 ± 2.34	1.345	0.197
Short - run low - gray - level		19.05 ± 3.34	18.34 ± 3.12	1.635	0.103

BMI, body mass index; VAS, visual analogue scale; GLCM, gray-level co-occurrence matrix.

### 3.3 Multivariate logistic regression analysis of functional outcomes after rotator cuff repair

**Figure 1:**
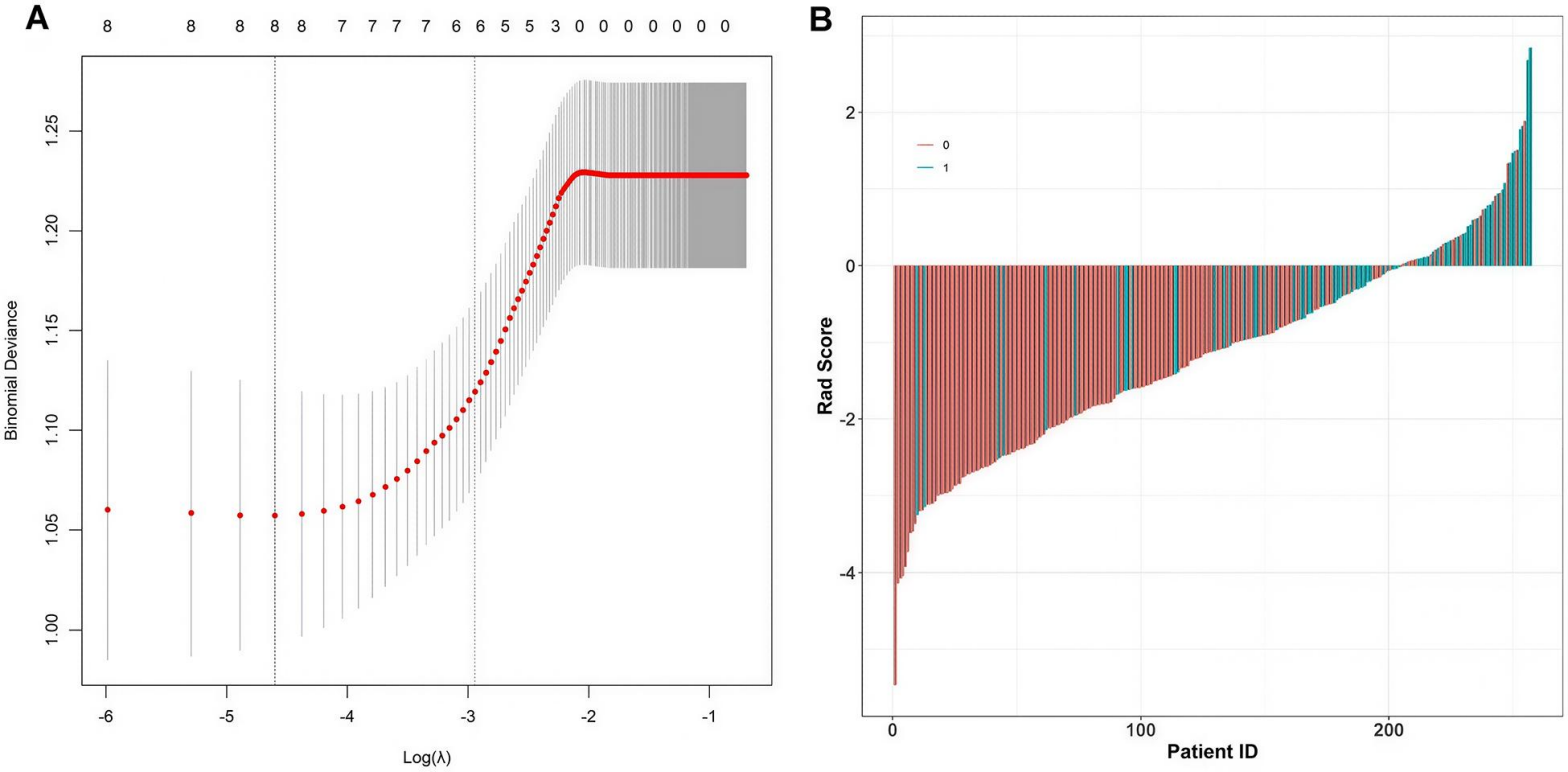
LASSO regression analysis **(A)** binomial deviance with regularization parameter and **(B)** patient risk score stratification.

Taking poor postoperative outcomes of patients as the dependent variable (1 = poor - outcome group, 0 = good - outcome group), the indicators with statistical significance in the univariate analysis were included in the LASSO regression for variable screening. Variables were selected using the screening criterion of lambda.min (Figure 1). The indicators retained were included in the multivariate Logistic regression analysis. The results showed that age, preoperative VAS, tear area, tear maximum length, tendon retraction distance, standard deviation of gray - scale, and GLCM entropy were risk factors for poor outcomes after rotator cuff repair (*P* < 0.05), while the preoperative Constant - Murley score was a protective factor (Table 3).

**Table 3:** Multivariate logistic regression analysis of poor outcomes after rotator cuff repair.

Factor	*β*	SE	Wald	*P*	OR	95%CI
Age	0.054	0.020	7.187	0.007	1.056	1.015–1.098
Preoperative VAS	0.255	0.118	4.656	0.031	1.291	1.024–1.627
Preoperative Constant - Murley score	−0.057	0.021	7.100	0.008	0.944	0.906–0.985
Tear area	0.018	0.005	11.212	0.001	1.018	1.007–1.029
Tear maximum length	0.103	0.028	13.405	0.001	1.109	1.049–1.172
Tendon retraction distance	0.209	0.062	11.221	0.001	1.232	1.091–1.392
Standard deviation of gray - scale	0.090	0.037	6.111	0.013	1.095	1.019–1.176
GLCM entropy	1.063	0.423	6.327	0.012	1.345	1.065–1.629
Constant	−12.384	2.703	20.991	0.001		

VAS, visual analogue scale; GLCM, gray-level co-occurrence matrix.

### 3.4 Construction of the nomogram prediction model for functional outcomes after rotator cuff repair

**Figure 2:**
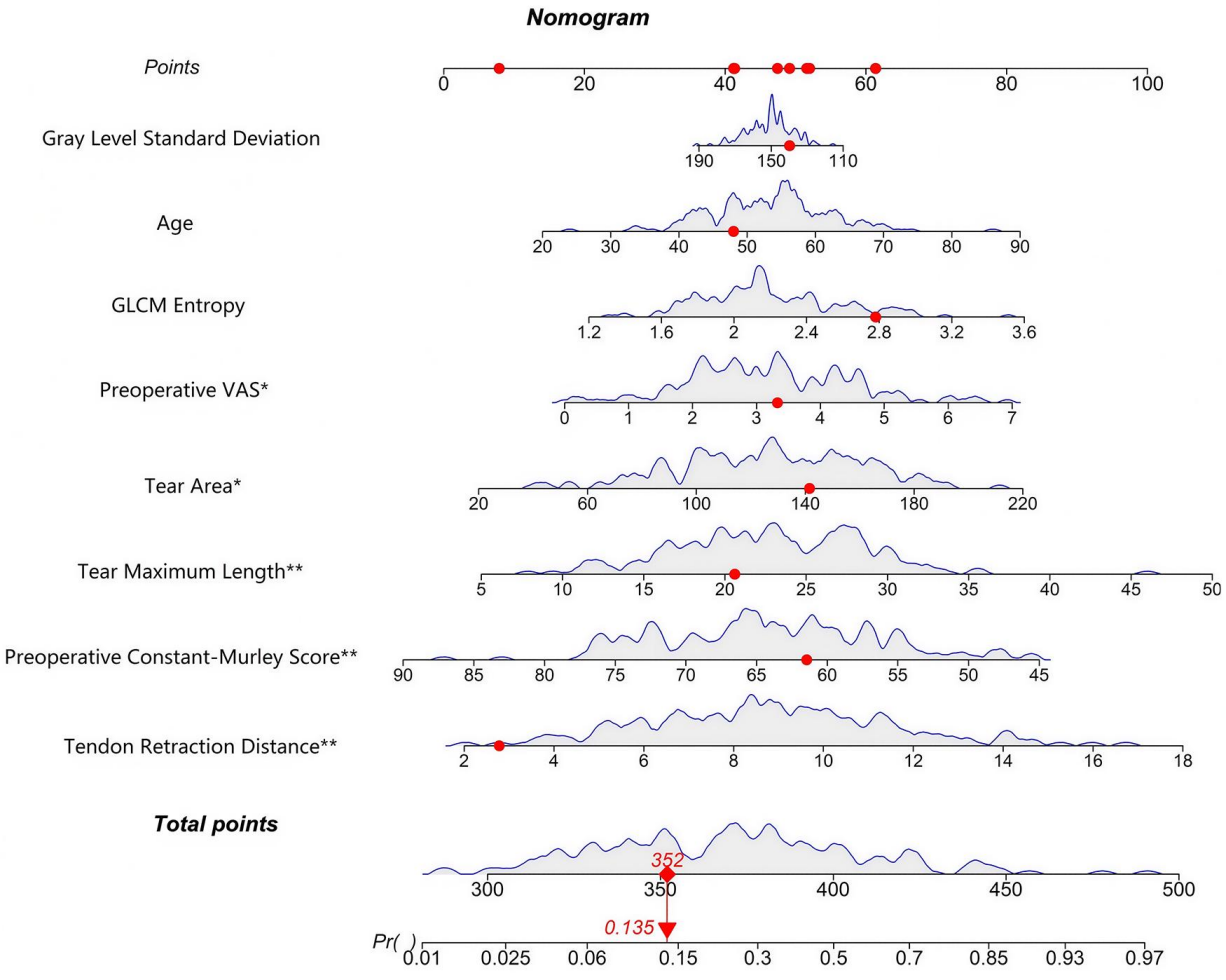
Nomogram for functional outcomes after rotator cuff repair. VAS, visual analogue scale; GLCM, gray-level co-occurrence matrix.

Based on the influencing factors screened by the multivariate logistic regression analysis, a nomogram prediction model was established to evaluate the functional outcomes after rotator cuff repair. Each independent risk factor in the model was assigned a corresponding score, and the total score associated with poor functional outcomes after rotator cuff repair could be calculated. The higher the score, the higher the predicted probability of poor postoperative functional outcomes, as shown in Figure 2. As illustrated in the nomogram, a representative patient was marked with red dots for each variable: Gray Level Standard Deviation = 150, Age = 50 years, GLCM Entropy = 2.8, Preoperative VAS = 3, Tear Area = 140 mm^2^, Tear Maximum Length = 20 mm, Preoperative Constant-Murley Score = 60, Tendon Retraction Distance = 2 mm. The total points of this patient were 352, corresponding to a predicted probability of poor postoperative functional outcome of 0.135. According to the optimal cut-off value of 0.433, this patient was classified as low-risk for poor functional outcome after rotator cuff repair.

**Figure 3:**
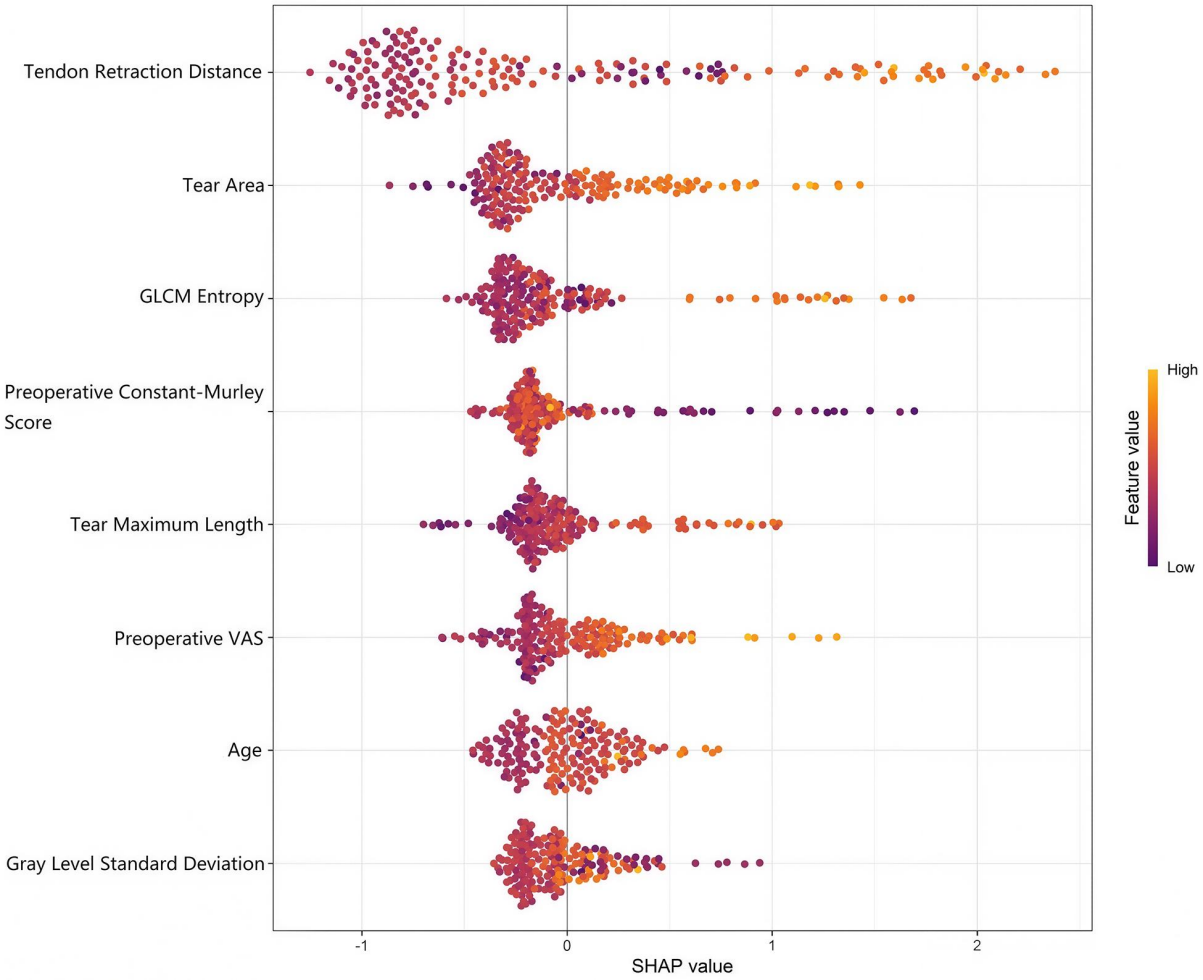
SHAP variable importance diagram. VAS, visual analogue scale; GLCM, gray-level co-occurrence matrix.

The SHAP variable importance diagram showed that tendon retraction distance, age, and preoperative VAS had relatively large impacts, as shown in Figure 3. The results of the collinearity diagnosis showed that the VIF of age, preoperative VAS, tear area, tear maximum length, tendon retraction distance, standard deviation of gray - scale, and GLCM entropy were 1.173, 1.026, 1.083, 1.140, 1.070, 1.108, and 1.115 respectively. This indicated that the problem of multicollinearity among the above variables was slight, and the degree of linear correlation among the independent variables was low. When constructing the regression model and conducting other analyses, the adverse effects of multicollinearity on model estimation and inference were relatively small, and the stability and interpretability of the model were well - guaranteed.

### 3.5 Evaluation and validation of functional outcomes after rotator cuff repair

**Figure 4:**
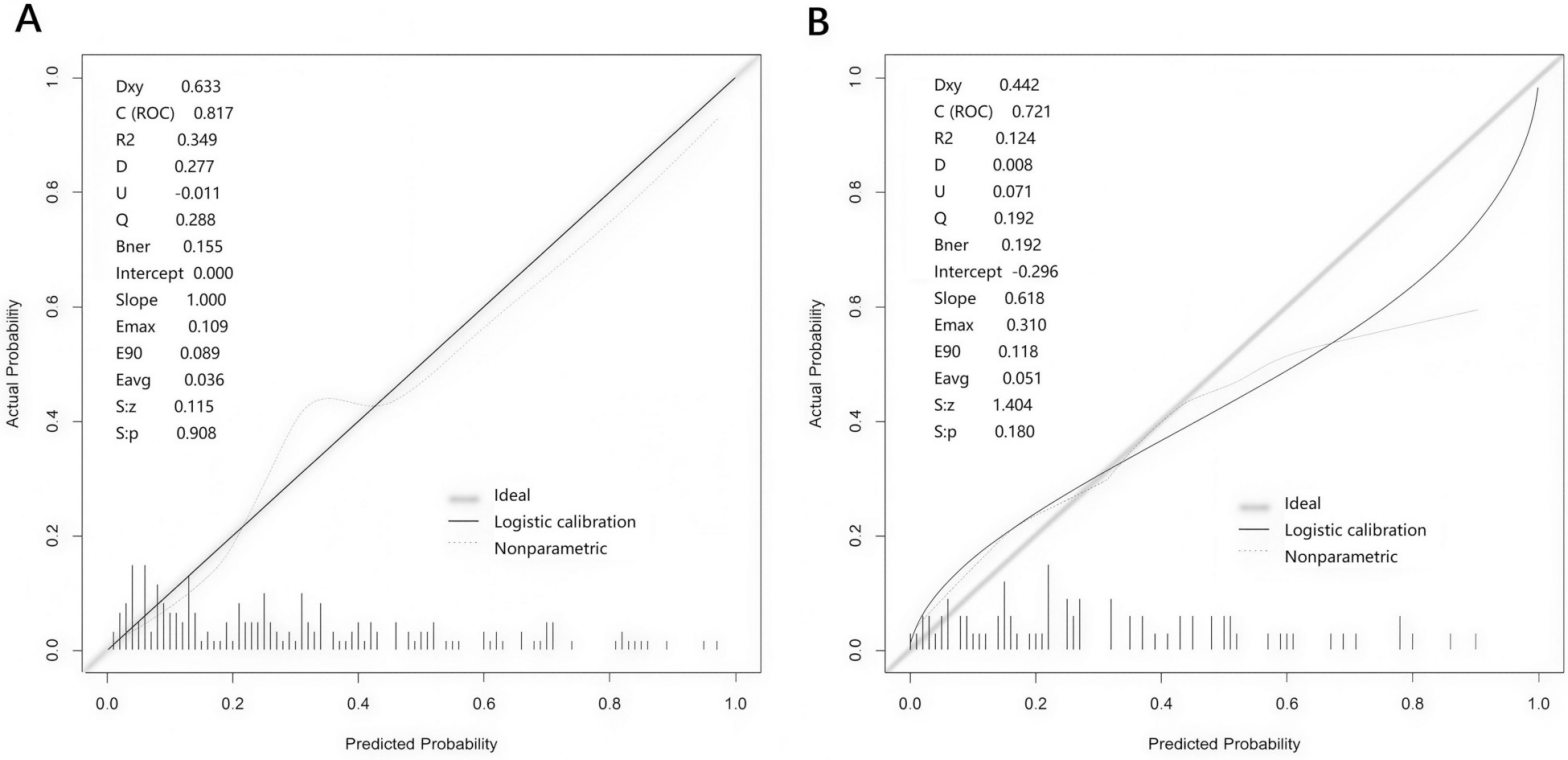
Calibration curves of the training set **(A)** and the validation set **(B)**.

**Figure 5:**
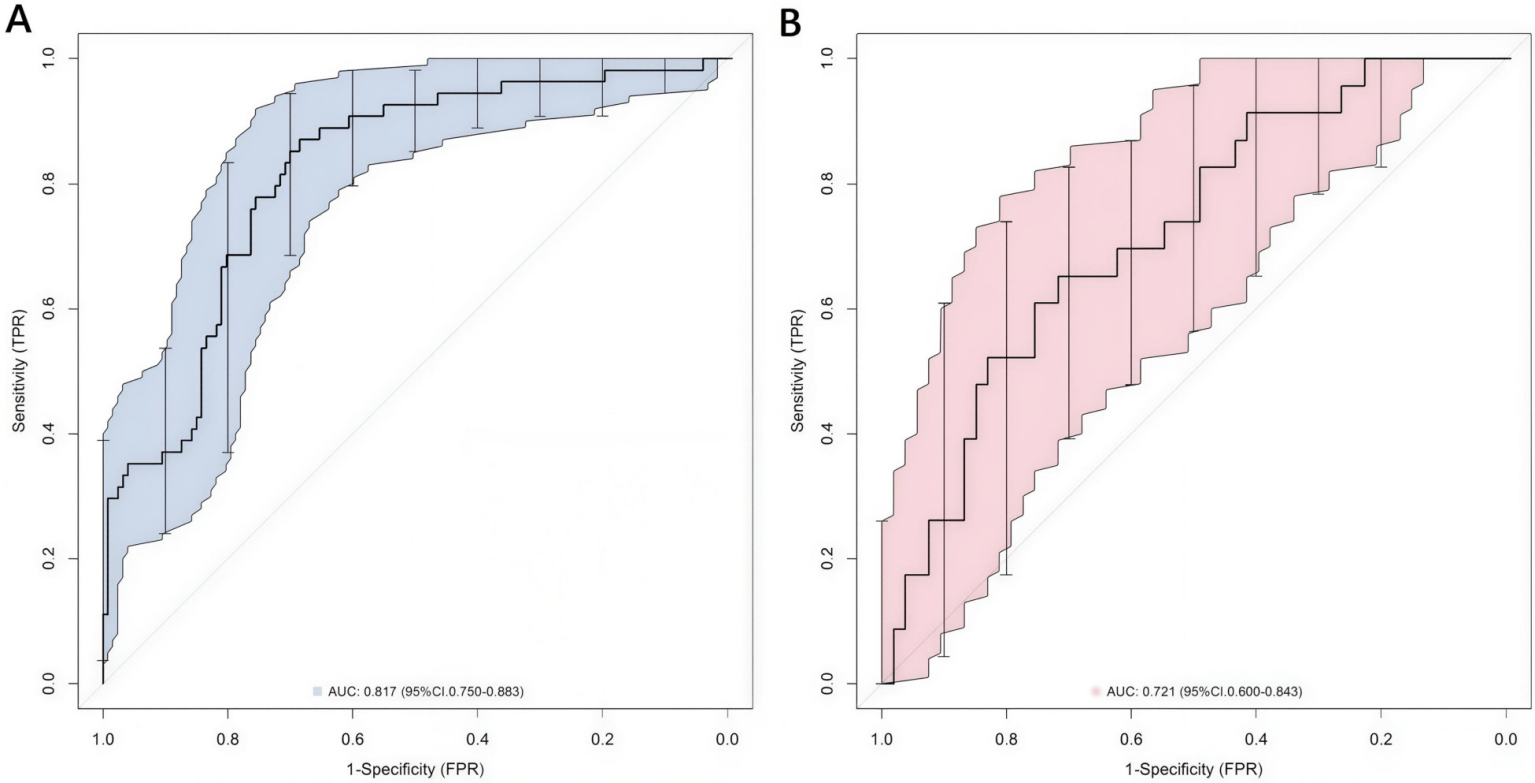
ROC curves of the training set **(A)** and the validation set **(B).**

In the training set and the validation set, the C - indices of the nomogram model were 0.817 and 0.721, respectively. The calibration curves showed that the mean absolute errors between the predicted values and the actual values were 0.155 and 0.192, respectively (Figure 4). The results of the Hosmer - Lemeshow test showed that the *χ*^2^ values of the two groups of data were *χ*^2^ = 7.722 (*P* = 0.504) and *χ*^2^ = 7.299 (*P* = 0.505), respectively. The ROC curves indicated that the AUC of the nomogram model for predicting postoperative functional outcomes in the training set and the validation set were 0.817 (95% CI: 0.750–0.883) and 0.721 (95% CI: 0.600–0.843), respectively (Figure 5). Delong test showed that there was no statistically significant difference in the AUC of the model between the training set and the validation set (*P* = 0.177). The sensitivities and specificities of the training set and the validation set at the cut-off value of 0.433 were 0.870 and 0.685, and 0.696 and 0.585, respectively. The model showed acceptable discrimination in the internal validation, yet its performance decreased significantly in the independent validation cohort (AUC reduced from 0.817 to 0.721), which indicated the insufficient robustness of the model. Its actual discriminative ability and generalizability in clinical practice need to be further verified by well-designed external multicenter studies.

### 3.6 Decision curve analysis of the model influencing postoperative functional outcomes

**Figure 6:**
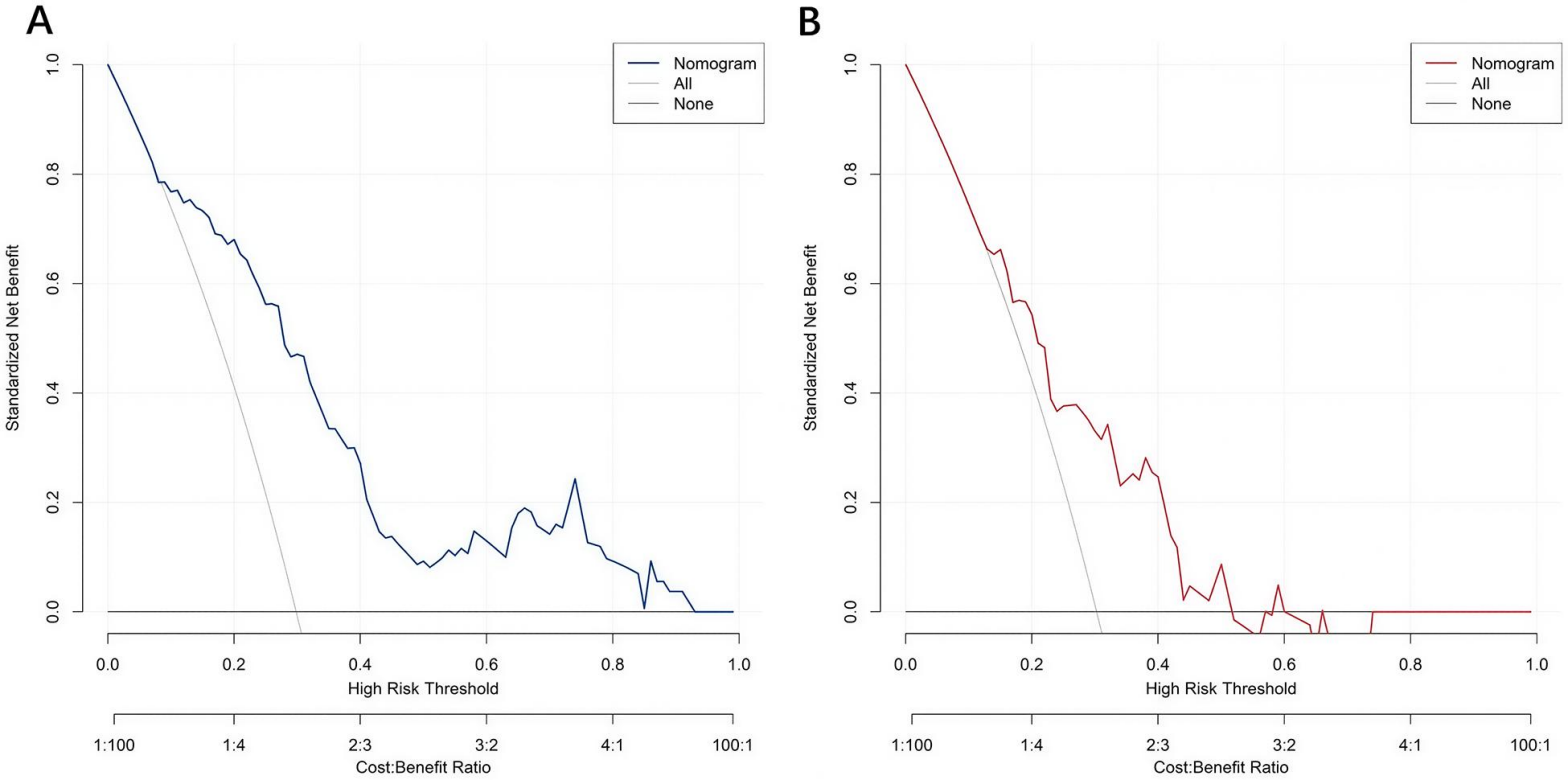
Decision curves of the training set **(A)** and the validation set **(B).**

The decision curve analysis showed that in the high - risk threshold range of 0–0.9, the standardized net benefit of the Nomogram curve of the training - set model was significantly better than that of the extreme strategies, demonstrating the decision - making value in the training stage. The validation - set model maintained a net - benefit advantage in the range of 0–0.65. This indicated that the model not only had clinical decision - making value in the training stage but also had good generalizability in the independent validation set, reflecting reliable clinical practicability and promotion potential (Figure 6).

## 4 Discussion

This study constructed a nomogram model integrating preoperative shoulder MRI radiomics features and clinical risk factors to predict 12-month functional outcomes after rotator cuff repair, overcoming the limitations of traditional single-indicator prognostic prediction. A total of 367 patients were divided into training and validation sets at a 7:3 ratio; through univariate analysis, LASSO regression feature selection and multivariate Logistic regression, we identified age, preoperative VAS score, tear area, longest tear diameter, tendon retraction distance, gray-scale standard deviation and GLCM entropy as independent predictors of poor postoperative outcomes, with the preoperative Constant-Murley score as a protective factor. The nomogram model constructed in this study shows potential clinical value in the internal validation cohort, which can help clinicians to preliminarily predict the risk of poor postoperative functional outcomes. However, the generalizability of the model to other populations or clinical settings remains to be confirmed.

From the perspective of the clinical and pathological significance of the predictive factors, the finding that age serves as an independent risk factor is consistent with clinical cognition. As age increases, the collagen synthesis capacity of the human rotator cuff tendon declines, the elasticity of the tendon tissue decreases, and the tensile strength reduces. Meanwhile, there is a decrease in muscle mass and an increase in the degree of fat infiltration. These physiological degenerative changes directly lead to a decline in the healing potential after tendon repair, and the postoperative functional recovery is naturally relatively slow (17–19). The predictive value of the preoperative VAS score highlights the important impact of the preoperative pain state on the prognosis. Patients with severe preoperative pain often indicate a more significant inflammatory response at the injury site. Chronic inflammation may indirectly impede postoperative functional recovery by disrupting the tendon repair microenvironment and impairing local blood circulation, which also confirms the importance of targeted preoperative pain management and inflammation control for improving the prognosis (20). The predictive efficacy of tear-related indicators including tear area, tear maximum length, and tendon retraction distance directly reflects the impact of injury severity on repair outcomes. The degree of tendon retraction is directly related to the difficulty of anatomical reduction during surgery. The greater the retraction distance, the smaller the contact area between the tendon and the bone bed, and the worse the stability of healing. Long - term retraction may lead to further degeneration of the tendon's own quality, thereby increasing the risk of postoperative re - tear. This finding also provides data support for focusing on the quality of tendon reduction during surgery (21, 22).

SHAP variable importance analysis further identified tendon retraction distance age and preoperative VAS as the top three core factors affecting postoperative functional outcomes. Tendon retraction distance ranks first because it directly determines the difficulty of surgical anatomical reduction and the stability of tendon-bone healing. Age is the second key factor as it is closely related to the degenerative changes of tendon tissue and the decline of healing potential. Preoperative VAS reflects the severity of local inflammatory response which affects the microenvironment of tendon repair. The clear ranking of core variables provides a clear direction for clinical decision making. Clinicians can conduct targeted preoperative assessment by focusing on these three indicators. Accurate measurement of tendon retraction distance by MRI is the primary preoperative evaluation content. For elderly patients or those with high preoperative VAS scores targeted intervention measures such as preoperative anti-inflammatory and pain relief can be taken in advance. This hierarchical assessment mode helps to optimize the individualized diagnosis and treatment plan for each patient. No counterintuitive findings were found in the SHAP analysis results. All variable importance rankings are consistent with the clinical cognition of rotator cuff injury and repair which also confirms the reliability of the prediction model constructed in this study.

The inclusion of radiomics features is an important innovation in this study. As independent predictors, the gray - scale standard deviation and GLCM entropy fully demonstrate the unique advantages of radiomics in mining microscopic pathological information. Traditional imaging evaluations remain largely at the qualitative description level and fail to quantify the microscopic structural changes of tendon tissue. The gray - scale standard deviation reflects the degree of dispersion of image gray - scale values and is closely associated with the heterogeneity of tendon tissue density. This density heterogeneity of rotator cuff tendons is mainly derived from edema fatty infiltration and collagen disorganization in the injured area (23, 24). Edema foci in rotator cuff tendons show high signal intensity on FS-T2WI and fatty infiltration foci present uneven gray-scale distribution while collagen disorganization destroys the uniform density of normal tendon tissue. The aggravation of these pathological changes will further increase the degree of density heterogeneity and the corresponding gray-scale standard deviation values. The GLCM entropy represents the complexity of image texture and can indirectly reflect the severity of pathological changes such as tendon fibrosis and fat infiltration (25, 26). This texture complexity of rotator cuff tendons is closely related to the mixed pathological patterns of collagen structural disorder focal fatty infiltration and interstitial edema (27). The disordered arrangement of collagen fibers breaks the regular texture of normal tendons and the interspersion of fatty infiltration and edematous areas in the tendon stroma further increases the irregularity of local tissue texture (28). Higher GLCM entropy values are indicative of more severe mixed pathological changes in rotator cuff tendons. These quantitative features enable objective capture of microscopic pathological information undetectable by traditional imaging, making up for the deficiency that clinical factors can only reflect the macroscopic state, enabling the prediction model to more comprehensively cover multi - dimensional factors affecting the prognosis. In addition, the preoperative Constant - Murley score as a protective factor indicates that patients with a better preoperative basic functional state possess better reserve functions of shoulder - related muscles and tendons and greater postoperative recovery potential. This result provides a clear reference for clinical screening of high - risk patients. For patients with a low preoperative Constant - Murley score, more intensive peri - operative intervention plans can be formulated in advance.

Currently, some studies have explored the prognostic evaluation after rotator cuff repair. *Longo* measured the anatomical parameters of rotator cuff tendons using MRI to provide references for diagnosis (8). *Cho* relied on deep learning of intraoperative arthroscopic images to predict re-tears (6). *Ding* constructed a re-tear prediction model based on lipoproteins with an AUC of 0.866, providing a metabolic perspective for re-tear risk assessment (12). We integrated preoperative MRI radiomics features with key clinical factors to predict the 12-month functional outcomes after rotator cuff repair, which can help clinicians quickly identify high-risk patients with poor postoperative functional recovery before surgery and reduce the threshold for clinical application through an intuitive scoring system. This study has several advantages. First, it realizes the integration of clinical risk factors and radiomics features. Clinical factors can reflect the overall health status of patients and the macroscopic background of the injury, while radiomics features reveal the microscopic pathological alterations at the injury site. Second, the constructed nomogram model has the advantage of visualization. It transforms the complex regression equation into an intuitive scoring system. Clinicians can rapidly evaluate the risk probability of poor postoperative outcomes for patients without complex statistical knowledge, with strong operational convenience and easier application in daily clinical practice.

Despite the above advantages, this study still has several limitations that need to be addressed. First, the study only conducted internal validation based on non-parametric Bootstrap sampling and lacked external multicenter validation, which is the most substantial limitation of this study. The model performance decreased significantly in the independent validation cohort (AUC reduced from 0.817 to 0.721), which suggests that the internal validation results have a certain optimistic bias, and the internal validation can only correct the overfitting bias to a certain extent and cannot reflect the actual performance of the model in different clinical centers and patient populations. Second, this is a single-center retrospective study with potential selection bias. The included patients are all from a single medical institution with relatively concentrated clinical characteristics surgical methods and rehabilitation protocols. The sample size is also relatively small which may lead to insufficient representativeness of the research population. This single-center nature also results in the lack of external validation for the constructed nomogram model. internal validation via Bootstrap sampling can only correct overfitting bias to a certain extent, and the lack of external validation is a major limitation of this study. The drop in AUC from the training set (0.817) to the validation set (0.721) suggests moderate optimism in the model's performance, and the generalizability of the model to other medical centers or patient populations cannot be confirmed. Third, the follow-up time is only 12 months after surgery. The functional recovery after rotator cuff repair is a long-term process. Some patients may experience functional fluctuations or retear after 12 months. Longer follow-up data are needed to assess the long-term predictive value of the model. Fourth, the extraction of radiomics features relies on manual delineation of the region of interest. There is still some subjectivity despite the guaranteed measurement reliability via intraclass correlation coefficient test. Fifth, postoperative rehabilitation intervention measures are not included in the predictive factors. The standardization and implementation of rehabilitation training have an important impact on functional recovery and may change the weight of each predictive factor. Sixth, Dichotomization of the Constant-Murley score at 80 may lead to some information loss compared with continuous or ordinal modeling, as it simplifies the continuous functional outcome into a binary variable. However, this approach is clinically meaningful and facilitates the interpretation of the prediction model for clinicians, allowing them to quickly identify patients at high risk of poor functional outcomes. Seventh, the study did not consider the influence of the specific types of rotator cuff injuries including isolated supraspinatus tear and multi-tendon combined tear, as well as associated injuries including acromioclavicular joint lesions and the long head of biceps tendon injury. These factors may also affect the postoperative outcomes and need to be further investigated in subsequent studies. Therefore, the biological and pathological correlates of the identified radiomics features (gray-level standard deviation and GLCM entropy) with rotator cuff tissue lesions are only explored in general terms. The specific associations between these radiomics features and pathological changes such as fatty infiltration, fibrosis and tendon degeneration of the rotator cuff have not been deeply analyzed, and this study failed to cite musculoskeletal-specific radiomics literature for targeted interpretation. The pathophysiological mechanisms underlying the correlation between these radiomics features and rotator cuff repair outcomes remain to be further verified in subsequent studies.

Corresponding solutions will be adopted in future studies to address the above limitations. For the single-center nature selection bias small sample size and lack of external validation multi-center prospective cohort studies will be carried out. More patients with different clinical characteristics and treatment modes will be included to expand the sample size and verify the external validity and generalizability of the model. For the possible overfitting problem machine learning algorithms with stronger generalization ability will be used to optimize the model structure and combine more external validation datasets to test the model stability. For the 12-month follow-up short-term follow-up will be extended to at least 24 months to explore the long-term predictive value of the model for postoperative functional recovery and retear risk. Deep learning technology will be applied to realize automatic segmentation of the region of interest to reduce the subjectivity of radiomics feature extraction and improve measurement accuracy. Postoperative rehabilitation related variables and specific injury types associated injuries will be included in the prediction model to further enrich the model variables and improve the comprehensiveness and accuracy of prognosis prediction.

## 5 Conclusion

The nomogram model integrating preoperative MRI radiomics features and clinical risk factors constructed in this study has potential to predict the functional outcomes 12 months after rotator cuff repair. Among them, tendon retraction distance, age, and preoperative VAS score are the key predictive indicators. This model demonstrates favorable discrimination, calibration, and clinical utility, which can help clinicians quickly identify high - risk patients with poor postoperative functional recovery before surgery, and then formulate individualized surgical plans and rehabilitation programs. For instance, more secure repair techniques can be adopted for high - risk patients, the rehabilitation training period can be extended, or preoperative pain management and inflammation control can be strengthened to ultimately improve the patients’ prognosis. In the future, multicenter, prospective studies are needed to verify the external validity of the model. Furthermore, deep - learning technology should be combined to optimize the radiomics feature extraction method, and more potential influencing factors such as postoperative rehabilitation should be included to further improve the predictive efficacy and clinical generalizability of the model, providing a more robust evidence - based medical basis for the precise treatment and prognosis assessment of rotator cuff repair.

## Data Availability

The raw data supporting the conclusions of this article will be made available by the authors, without undue reservation.
